# Back to the Future: A Glance Over Wolf Social Behavior to Understand Dog–Human Relationship

**DOI:** 10.3390/ani9110991

**Published:** 2019-11-18

**Authors:** Giada Cordoni, Elisabetta Palagi

**Affiliations:** 1 Natural History Museum, University of Pisa, Via Roma 79, 56011 Calci, Pisa, Italy; giada.cordoni@unipi.it; 2 Ethology Unit, Department of Biology, University of Pisa, Via Volta 6, 56126 Pisa, Italy

**Keywords:** social tolerance, social attentiveness, reconciliation, consolation, appeasement, play fighting, leverage, behavioural plasticity, *Canis lupus lupus*

## Abstract

**Simple Summary:**

Wolves, the ancestors of dogs, are one of the most cooperative canine species. This cooperative propensity derives from the fact that each subject needs other group members to obtain resources and increase survival. The pack functions as a unit in which each individual collaborates in territory defence, hunting, and rearing of offspring. For this reason, even though a clear hierarchy exists among wolves, subordinates can provide help to dominants to obtain social tolerance in a sort of commodity exchange. Wolves can make peace after aggression, console victims of a conflict, and calm down the aggressors. This set of behaviors, also called post-conflict strategies, requires a social attentiveness towards others’ emotional state and the ability to coordinate appropriate reactions. Adult wolves also play. They engage in play fighting, which strongly resembles real fighting, by finely modulating their motor actions and quickly interpreting playmates’ *intentions*, thus maintaining the non-serious playful mood. All these cognitive and social skills were a fertile ground for the artificial selection operated by humans to redirect the cooperative propensity of wolves towards dog–human affective relationship.

**Abstract:**

This review focuses on wolf sociobiology to delineate the traits of *cooperative baggage* driven by natural selection (wolf-wolf cooperation) and better understand the changes obtained by artificial selection (dog-human cooperation). We selected some behaviors of the dog’s ancestors that provide the basis for the expression of a cooperative society, such as dominance relationships, leverage power, post-aggressive strategies, and playful dynamics between pack members. When possible, we tried to compare the data on wolves with those coming from the dog literature. Wolves can negotiate commodities when the interacting subjects occupy different ranking positions by bargaining social tolerance with helping and support. They are able to manage group disruption by engaging in sophisticated post-conflict maneuvers, thus restoring the relationship between the opponents and reducing the spreading of aggression in the group. Wolves engage in social play also as adults to manipulate social relationships. They are able to flexibly adjust their playful interactions to minimize the risk of escalation. Complex cognitive abilities and communicative skills are probably the main proximate causes for the evolution of inter-specific cooperation in wolves.

## 1. Introduction

*“Domestication is a sustained multigenerational, mutualistic relationship in which one organism assumes a significant degree of influence over the reproduction and care of another organism in order to secure a more predictable supply of a resource of interest, and through which the partner organism gains advantage over individuals that remain outside this relationship, thereby benefitting and often increasing the fitness of both the domesticator and the target domesticate”*.

[1]

Even though we intuitively understand what domestication is, there is a surprising lack of consensus on its definition. Beyond the agreement that domestication involves a relationship between a *domesticator* and a *domesticated organism*, there are many debates on what this relationship entails and how and when it occurs. Many definitions of this process take into account only the perspective of the *domesticator*, emphasizing the impact of humans in this role. Historically, humans deliberately and opportunistically select a wild species for creating its “domesticated counterpart” of whom they control all aspects of its life cycle [1]. According to “*domestication syndrome*” [2], a set of morphological, physiological, reproductive, and behavioral traits can be observed in domesticated species but not in their wild ancestors. In animals, these traits can include, for example, increased fecundity, altered coat color, reduced body size, facial neoteny, increased docility, and hypersociability [3,4,5].

Dog, the oldest domesticated animal by humans, certainly shows many traits of the “*domestication syndrome*” such as, reduced body size and snout length and increased docility, tameness, and playfulness [5,6]. Many researchers suggest that the beginning of dog domestication took place in the Early Upper Paleolithic (~30,000 years ago), when people still lived in small groups as hunter–gatherers and agriculture was not yet practiced [7,8,9,10]. Nevertheless, the fossil remains confidently indicate the appearance of dog in Europe ~15,000 years ago [11]. Basing on morphological and genetic analyses, wolves (*Canis lupus lupus*) are undoubtedly the ancestors of modern dogs [9,11,12,13]; while the wild wolf phenotype changed markedly, the genotype changed only minimally, leaving domestic dogs, genetically speaking, still as wolves [10]. The domestic relationship between people and dogs is the result of a wolf ecological strategy to cope successfully with the Late Pleistocene environmental changes due to the increased human population. The plasticity characterizing wolves permitted them modifying their ecological niche by joining the human niche; people possibly facilitated this change by incorporating some young wolves into their groups and by selecting over time the more docile and tameness subjects [10,13,14]. 

According to the *Domestication Hypothesis*, it has been proposed that through an evolutionary and ontogenetic positive feedback processes, dogs have become more socially tolerant and attentive than wolves, two characteristics that are crucial for cooperation to occur [15,16]. However, the studies supporting the *Domestication Hypothesis* were mainly based on wolf–dog behavioral difference in relation to their interactions with humans [15,16,17,18,19,20,21]. Range & Virányi [20,21] proposed an alternative (but not exclusive) theory, the *Canine Cooperation Hypothesis*, according to which these differences may reflect only an improved capacity of dogs to accept humans as social partners instead of an increased in their general tolerance, attention, and cooperation degree. Some cognitive studies have shown that wolves can attentively use the information provided by a familiar human to solve a task [22], can follow human gaze as readily as conspecific gaze [23] and are more successful than dogs in copying the actions of conspecifics [24,25]. Moreover, when wolves and dogs (reared under the same conditions) were faced with a series of object-choice tasks, wolves showed similar results to dogs in responding to communicative and behavioral cues, but they outperformed dogs in their ability to follow causal cues [26]. All these authors suggested that high level of cooperativeness characterizing wolf society may come together with a high propensity to pay close attention to others’ actions. Also, by moving the focus from human–wolf to wolf–wolf interactions, researchers have highlighted the high cooperativeness and cohesiveness characterizing wolf packs [20,26,27,28].

This review aims at delineating the possible pathways of the behavioral changes that, over the time, have led from wolves to dogs and, consequently, to the strong dog-human relationship. To address this issue, we deal with different aspects of wolf sociality. In particular, we “take a glance” to dominance, post-aggressive and playful dynamics between pack members by comparing the findings on wolves with those on dogs [29]. Can the wolf-dog behavioral difference be credited only to the domestication process? Can the domestication process have induced a shift of social tolerance and attentiveness from conspecifics to humans, thus leading dogs towards an exclusive inter-specific cooperation?

## 2. Social Tolerance by Dominants, Leverage Power by Subordinates, and Peaceful Strategies by All Group Members

Wolf pack is defined as a cohesive family group, including a long-term bond breeding pair, mature offspring, and pups; occasionally, an unrelated individual may join the group [30,31]. All wolves participate in pack life by creating a system of division of *labour* in which individuals cooperatively hunt and defend their territories and collectively rear the pups [30,32]. The socialization begins at around four months of age when cubs start to follow the adults on hunting trips (“hunting school”). During this period, puppies improve their motor and perception skills and perfect mutual interaction and coordination with conspecifics. The affinitive relationships develop during puberty, when maturing individuals are slowly integrated into the daily life of the group [30,31,32]. The strict social association between pack members finds support in a study of Cassidy and McIntyre [33]. The authors recorded 121 territorial inter-pack conflicts in Yellowstone National Park, and in 17.6% of cases, wolves engaged in aggression to defend their pack fellows.

Within the pack, puppies generally occupy lower ranking positions compared to their parents and older siblings. When wolves reach sexual maturity (~2 years), they disperse from their natal group, attempt to pair with other dispersed wolves and start their own packs, thus avoiding competing for dominant-breeder *status* with natal group members [30,32]. However, under some conditions, both in the wild and in captivity, mature individuals delay dispersal or do not disperse at all; in these cases, competition for dominant-rank may be stronger [32,34,35]. In captive packs, wolves often have a linear hierarchy in which all males are dominant over females [32,36,37]. Nevertheless, the more appropriate term to define dominance relationships in a typical wolf pack (nuclear, extended or complex families) is “age-graded dominance hierarchy” [31,36]. Moreover, the subordinate individuals can sometimes oppose their leader’s actions; for this reason, Zimen [38] defined the leadership in wolf packs as a “qualified democracy”, in which no subject decides alone to carry out activities that are crucial for the group survival. Recently, Range and colleagues [21] found that, under feeding conditions, captive wolves are more tolerant compared to dogs. Indeed, high-ranking dogs monopolized the resources while low-ranking individuals showed deference by staying apart without trying to obtain food from the dominant subjects. Conversely, subordinate wolves overtly challenged the dominant ones to subtract food from them. Wolves are cooperative hunters [34] and, in term of mutual beneficial exchanges, all members of the pack have the possibility to access food, independently of their ranking position. On the contrary, domestic dogs rely on humans for food and feral dogs are solitary scavengers, hence, both do not depend on conspecifics’ support for obtaining the resource [39]. These findings suggest that, in the wolf society, the power is not entirely “in the hands” of the physically stronger subjects. The subordinates can exert leverage power [40,41] because of their support to the pack life and their cooperation is gained by high-ranking individuals through peaceful sharing instead of aggressive coercion [42].

Nevertheless, despite this cooperating social system, the presence of aggression is the other inevitable side of the coin that leads to a temporary interruption of the inter-individual relationships [29,43]. To cope with aggression and the consequent social damage, as it occurs in many social mammals (human primates [44]; non-human primates, [45,46,47,48,49]; dolphins [50]; spotted hyenas, [51]; red-necked wallaby [52], wolves engage in post-conflict contacts such as reconciliation (i.e., the first affinitive contact exchanged by the former opponents relatively shortly after a conflict [53]).

By analyzing 3344 conflicts, Cordoni and Palagi [37] provided the first evidence for the occurrence of reconciliation in wolves by observing the pack hosted at the Pistoia Zoo (Italy), which was categorized as a “disrupted family” due to the absence of the alpha female. The high level of conciliatory contacts was uniformly distributed across the different sex–class combinations. Interestingly, reconciliation was not linked with rank distance between opponents but it positively correlated with coalitionary support (defined as a third party joining an ongoing conflict by attacking one of the opponents in support of the other, [54]). Generally, in social mammals, the high level of support can unveil high level of cooperation. In wolves, alliances [55] and reconciliation [37] act as diffuse non-dispersive mechanisms that concur in strengthening group cohesiveness. The occurrence of reconciliation was confirmed by other studies both in wild [56] and captive wolves [57]. Also, in the wild condition, wolves showed high level of conciliatory contacts and, once more, the finding was explained by the authors in the light of the strong cohesion between pack members [56].

Even though few doubts remain for the occurrence of reconciliation in wolves [37,56,57], contrasting results derive from canine reconciliation. Cools and co-workers [58] demonstrated reconciliation in small groups of dogs sharing a pen. Recently, Cafazzo and colleagues [57] by studying four captive small packs of dogs and wolves provided evidence for reconciliation in wolves, but not in dogs. Indeed, in this study, the dogs avoided affiliating with their opponents after conflicts. The social repairing function ascribed to the reconciliation mechanism [53] is probably useless for dogs. The difference in socio-ecological habits between dogs and wolves may cause the difference in their conciliatory tendency. In dogs, the absence of cooperative hunting and collective rearing of offspring limits the need to maintain friendly and peaceful social relationships with conspecifics [57].

Beyond reconciliation, other types of post-conflict interactions can occur. Group members not involved in aggression (bystanders) can spontaneously offer friendly contacts to both victims (“consolation”; [53]) and aggressors (“appeasement”; [59]). Even though the cognitive and emotional skills underpinning these contacts are still under debate, “consolation” and “appeasement” seem to serve different functions.

**Figure 1:**
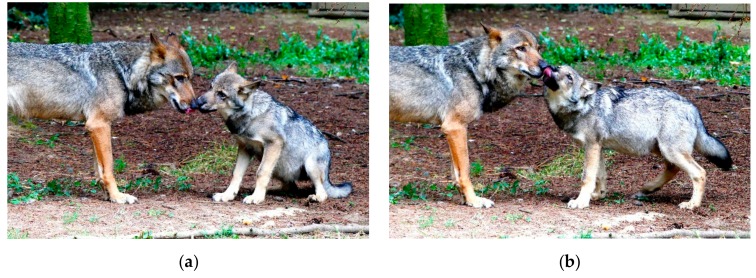
Reciprocal muzzle licking during a post-conflict triadic affiliation (“consolation”) between an adult (the consoler) (**a**) and an immature subject (the victim) (**b**) Photos by Elisabetta Palagi.

In wild and captive wolves both “consolation” and “appeasement” are present [43,54,56]. In the Pistoia pack [43,54], the two post-conflict behavioral strategies occur within two minutes after the end of the aggression and are performed with comparable levels. Despite these similarities, “consolation” and “appeasement” seem to play different roles. In the Pistoia wolves, in about 45% of cases, bystanders offer affinitive contacts to aggressors, which generally occupy high-ranking positions. Such calming interactions have the immediate effect to reduce the likelihood of renewed aggression toward other group members by the previous aggressor. On the other hand, affiliation that bystanders direct towards the victims follows the relationship quality linking the subjects more than their hierarchical positions: the stronger the bonding, the higher the frequency of affinitive contacts. Furthermore, these contacts protect the victim against the reiterated attacks from the previous aggressor. In sum, “consolation” may represent a “victim protection” strategy and “appeasement” a “bystander protection” strategy, thus highlighting the functional dichotomy characterizing these two behaviors [43,54]. The Figure 1 illustrates a reciprocal muzzle licking during a post-conflict triadic affiliation also named “consolation”.

Although bystander post-conflict affiliation has been demonstrated in dogs [58], it does not seem that familiarity between interacting subjects significantly affects the behaviour. Cools and colleagues [58] showed that bystander affinitive contacts were more frequently directed to victims than to aggressors. Moreover, victim-directed affiliation was markedly higher than affinitive interactions between the former opponents. The scarcity of data did not permit the authors to investigate the possible functions of dog “consolation” and “appeasement” leaving the question still open.

## 3. The Playful Wolf: Tactics and Plasticity for a Deeper Knowledge of Others

Beyond dominance, affiliation, and conflict management, social play is another type of interaction that can affect the social dynamics within a group [60]. Immature individuals of many mammalian species engage in different types of playful activities [61]. The pervasive distribution of play suggests that the core neural circuitry underpinning the modulation of this behavior may have evolved early in mammals and it may be shared by different species [62]. 

During play, animals execute motor action patterns that are recruited from “serious” contexts such as agonistic, anti-predatory and sexual [61]. However, these patterns are performed in an exaggerated, incomplete, repeated, mixed and unexpected manner [63,64,65].

The first playful experience occurs between mothers and infants [66]; these sessions represent a good training ground for preparing infants to the future interactions with peers [67,68]. During ontogeny, play concurs in implementing physical, cognitive, emotional, and social skills of individuals by guaranteeing a safe and fruitful “environment” in which making training [45,63,69,70,71,72,73]. Held and Špinka [74] suggested that play could provide individuals with psychological benefits by promoting opioid-mediated pleasurable experiences: the better individuals feel, the more individuals play. The resultant positive feedback between play and animal well-being becomes an important tool for improving and ameliorating the management of animals in captivity. 

Although benefits provided by social play can vary depending on context (e.g., pre-feeding, mating period), habitat, playmate characteristics (e.g., sex, age, and rank) and species sociality (e.g., tolerant versus despotic), the intrinsic nature of play requires an implicit agreement, cooperation, and negotiation between players, who have to trust one another [65,75,76,77]. Playmates reach this goal by fine-tuning their contact interactions and by avoiding the performance of behaviors that might be misinterpreted. By playing fairly, animals may acquire the social competence and rules that are at the basis of a peaceful coexistence [60].

Peaceful cohesiveness is a feature of wolf society that is guaranteed also by playful activity [54,60,78]. Although most studies have focused on play in domestic dogs [6,79,80,81], this behavior does not appear as an artefact of domestication since also wolves play even during adulthood [78,82].

It has been suggested that, through play, adult subjects can evaluate and manipulate the social relationships with group-members [71]. Cafazzo and colleagues [82] investigated play behavior in four captive wolf packs, two composed by immature peers and the other two by mixed-age subjects (puppies and adults). A positive linkage between play frequency and relationship quality was found; indeed, those dyads that spent more time in relaxed play (defined as play sessions involving a limited number of offensive patterns) engaged in more interactions belonging to the affinitive domain. Moreover, in mixed-age groups, but not in peer groups, the frequencies of aggression are negatively correlated with play levels. Interpreting this result, the authors suggested that play can limit aggressiveness between group-members only after the establishment of clear hierarchical positions.

The observation of the wolf pack hosted at the Pistoia Zoo (Italy) led to contrasting results compare to those of Cafazzo and colleagues [82], although direct comparison can be misleading due to the different conditions under which the wolves were reared. In the Pistoia group, composed by adult related individuals, playful activity was not affected by both social relationship quality and aggression level, but it was strictly linked to dominance. Play was negatively correlated to the rank distance between fellows; in other words, subjects with closer ranking position played more than subjects differing greatly in rank [78]. In another study on the same pack, Cordoni and Palagi [54] compared the level of aggressiveness and steepness of hierarchy in two different periods (Sample 1 and Sample 2). During Sample 1, two adult females died and this event probably provoked delayed social effects which were manifest during the Sample 2 characterized by higher aggressiveness and steeper linear hierarchy. The re-arrangement of the dominance relationships within a pack occurs gradually and it is usually manifest later in time respect to the occurrence of the perturbing event (e.g., the removal of particular subjects; [83]). Intriguingly, in the Pistoia pack, the hierarchical difference between the two Samples had a significant effect on playful but not on affiliative dynamics. During the riskier period (Sample 2), wolves consistently reduced their playful activity, avoided playing during high-tension contexts (e.g., pre-feeding time) and limited the number of players per session by preferring dyadic (only two players) at polyadic interactions [54]. As a whole, these findings suggest that “rank rules dictate play rules”. By playing, wolves can acquire information about physical and cognitive skills of fellows with whom they can compete in the future for dominant position. Nevertheless, in order to maintain a “not serious” mood, wolves had to manage play in a flexible manner by place the interaction in the right time, in the right context, and with the right players [45,54,72,73,78].

Pal [84] observed 24 free-ranging dog puppies from birth to 13 weeks of age. In each litter, there were particular subjects that initiated offensive play more often than the other siblings did. Moreover, the same-sex offensive play was the predominant interaction between puppies. These findings suggest that, starting from five weeks of age, free-ranging dogs can employ playful activity to acquire information about individuals with whom they may compete in the future for reaching dominance positions, as it occurs in wolves [84].

In domestic dogs, adult-adult play does not follow the 50–50 rule that is a balance between offensive (“dominant”) and defensive (“subordinate”) patterns exchanged by playmates during the interaction. In play, adult dogs seem to hold the same dominance position they have outside the playful context [79]. Also, dog puppies do not adhere to the 50–50 rule by performing offensive playful patterns much more frequently than defensive ones [80]. Play becomes more asymmetrical as the puppies matured. Along with dog developmental pathway, the winning and the losing positions during playful interactions mirror the dominance relationships between individuals. Intriguingly, Cordoni and colleagues [6], by studying 49 domestic dogs in an off-leash dog-park, evidenced that the level of play asymmetry did not differ between “friends” (i.e., dogs that lived together or regularly interacted) and “strangers” (i.e., dogs that have never interacted before the observation). In the light of this result, dog play can be not only predictive of the dominance relationships between playmates, but it may serve an important function in maintaining good social bonds with specific partners. Nevertheless, independently of the inter-individual relationship, the playful sessions characterized by high asymmetry, and, consequently, by high competition, had a shorter duration compared to the more balanced sessions. The decrease in play duration and the use of clear communication can represent strategies used by dogs for overcoming the risk of escalation during very asymmetric playful interactions [6].

In wolf puppies, social play seems to be well-balanced with both immature partners performing a similar amount of self-handicapping behaviors, this reciprocity decreases when one of the players is an adult. When play involves two mismatched wolves, the session generally becomes more asymmetric [85]. The flexibility in managing the playful arousal is evident in the study by Cordoni & Palagi [54]. The adult wolves of the Pistoia group changed their play modality according to the period of observation. In Sample 1, the period characterized by low level of hierarchical steepness and aggressiveness, subjects engaged in more self-handicapping and role reversal manoeuvres, thus making their playful sessions more symmetric. This suggests that animals are able to flexibly adjust their playful tactics according to the social circumstances and that the play asymmetry is not always predictive of the dominance status of the players. In this view, the analysis of some social factors such as the exact quantification of (i) the hierarchical steepness, (ii) the level of affiliation and (iii) the bidirectionality of agonistic conflicts is mandatory before beginning a study on play in adult wolves.

## 4. Conclusion

*“Can the wolf-dog behavioral difference be credited only to the domestication process? Can the domestication process have induced a shift of social tolerance and attentiveness from conspecifics to humans thus leading dogs towards an exclusive inter-specific cooperation?”*

Through a systematic comparison between data coming from the available studies on wolves and dogs we propose possible answers to these questions.

A group of wolves moves as a unique entity, with each subject relying on others’ support to gain benefits and increase survival and fitness. In this network of cooperating individuals, some are dominants and some subordinates. These hierarchical relationships are not based on a mere deference of subordinates towards the dominants but on an exchange of services that both counterparts seem to put in act. In the wolf cooperative system, the social tolerance provided by dominants to subordinates can be repaid by subordinates with their help and support in the group maintenance activities. This does not necessarily mean that individuals cannot use their high-ranking position to obtain priority in certain domains. Indeed, a dominant can exert a strong control over resources to the detriment of subordinates. However, keeping everything under control through threats and overt aggression is energetically demanding, and, for this reason, trading can be also present when coercion by dominants is possible. In these situations, also the subordinates can exert their little amount of power over the dominants, that is called leverage. This delicate equilibrium permits and sustains the development of cooperation in social groups.

A further prerequisite for the development of a cooperative society is social attentiveness, which occurs when a subject is sensitive to others’ behaviors and needs. The level of attentiveness can change according to different factors such as contexts, kinship, relationship quality shared by the interacting subjects. When an individual is attentive to fellows, he/she can adjust and coordinate his/her competitive or cooperative reactions. The link between attention and action coordination appears clear in the “consolation” and “appeasement” dynamics occurring after a conflict. The ability to perceive the emotional mood of the victim (anxiety) or the aggressor (arousal) is highly beneficial to the subject who can reduce, through the post conflict affiliative interaction, the probability of further aggression thus concurring in the maintenance of group cohesion.

An important mix of cooperation and social attentiveness is mandatory during play fighting, an activity which is complex to manage and that can lead easily to misinterpretation. Animals have to finely read and rapidly interpret each single pattern of the playmate in order to react in an appropriate manner. This needs a profound knowledge of the partner, because the reaction can change as a function of the relationship linking the two players. This is a highly cognitive demanding behavior which comes into place when animals are strongly motivated to cooperate.

In conclusion, the domestication process has acted over *cooperative baggage* already present in the sociobiology of the dog’s ancestors. In wolves, such *baggage* has been driven by natural selection towards conspecifics, while in dogs, this *baggage* has been redirected by the artificial selection towards humans.
